# Aggregation-induced stabilization of pheophorbide, a water-soluble chlorophyll derivative

**DOI:** 10.3389/fnut.2026.1847974

**Published:** 2026-05-15

**Authors:** Yixiao Liu, Yishuang Liu, Yangbin Wang, Shuyu Wang, Shuyi Shi, Fangwei Li, Mingyong Zeng

**Affiliations:** 1 State Key Laboratory of Marine Food Processing and Safety Control, College of Food Science and Engineering, Ocean University of China, Qingdao, China; 2 Sanya Institute of Oceanography, Ocean University of China, Sanya, China; 3 College of Food Science and Nutritional Engineering, China Agricultural University, Beijing, China; 4 National Engineering Research Center for Fruit and Vegetable Processing, Ministry of Science and Technology, Beijing, China

**Keywords:** aggregation, chlorophyll, pheophorbide, stability, water solubility

## Abstract

**Background:**

Chlorophyll (Chl) is a valuable natural pigment, but its application is limited by poor water solubility and photoinstability. This study focuses on the preparation of pheophorbide (Phide), a water-soluble chlorophyll derivative, and investigates the possible role of molecular aggregation in enhancing its photostability.

**Methods:**

Phide was synthesized from Spirulina-derived chlorophyll through saponification. Structural characteristics were analyzed using Fourier Transform Infrared (FTIR) and UV–Vis spectroscopy. A concentration determination method was established by combining High-Performance Liquid Chromatography (HPLC) with UV–Vis spectroscopy. Photostability was evaluated through a 6-day light exposure experiment. In addition, Independent Gradient Model (IGM), Electrostatic Potential (ESP), and hole–electron analyses were performed on a parallel-stacked dimer model to explore the intermolecular interactions and electronic characteristics of the aggregated state.

**Results:**

Phide exhibited good water solubility and concentration-dependent aggregation behavior in aqueous solution. After 6 days of light exposure, the retention rate of high-concentration Phide reached 63.30%, which was significantly higher than that of the low-concentration system. Spectroscopic characterization and theoretical calculations suggested that Phide molecules tend to adopt a parallel stacking arrangement during aggregation. Van der Waals and electrostatic interactions were identified as major driving forces for aggregate formation. Hole–electron analysis further indicated the presence of charge-transfer characteristics and enhanced electronic delocalization within the aggregated structure.

**Conclusion:**

The experimental and theoretical results consistently suggest that molecular aggregation is associated with improved photostability of Phide in aqueous systems. This work provides insight into the aggregation behavior and photostability of dephytylated chlorophyll derivatives and offers a possible explanation for aggregation-associated stability at the electronic level. The findings may provide a useful basis for developing more stable water-soluble chlorophyll-derived colorant systems.

## 1 Introduction

Chlorophyll (Chl) is a type of natural green pigment found in green plant cells and is widely distributed in nature (1). Nevertheless, Chl’s structure makes it a lipid-soluble pigment. It is currently employed in a relatively limited manner, with the exception of its use in oil and cans as a natural pigment (2, 3). Its hydrophobic phytyl tail make Chl insoluble in water but easily soluble in oil (4), limiting its use in food industry. Meanwhile, Chl is sensitive to heat, light, oxygen, acid, and enzymes, leading to degradation and color change in food components (5, 6). This makes it challenging to maintain the original color and stability during processing. To address the water insolubility of chlorophyll, removing the phytyl chain and preparing metal chlorophyll derivatives, such as copper and zinc derivatives, can be effective strategies (7, 8). These derivatives can be produced during hot food processing or synthesized artificially. The most popular is Sodium Copper Chlorophyll (SCC), a water-soluble Chl derivative without a phytyl chain that changes central Mg ion to Cu atom and has a green color like native Chl. It is extensively used as a culinary coloring and deodorant in ice cream, candies, biscuits, and sweets (9). Nevertheless, the safety of synthetic colorants remains a significant concern for consumers. The FDA has identified a range of adverse effects associated with artificial colorants, including carcinogenesis, obsessive-compulsive disorder, sleep disturbances, irritability, hyperactivity, aggression, and inflammation (9–11). Copper and zinc are not macro elements required by the human body (12) and long-term intake of copper ions by the human body will cause a large amount of copper elements to accumulate in the liver, leading to copper poisoning.

Phide is a natural water-soluble Chl derivative with active function similar to Chl (13). Its inherent capacity as a natural photosensitizer enables synergistic integration with photodynamic therapy (PDT), demonstrating broad-spectrum anticancer efficacy through light-activated mechanisms (14). Phide has antibacterial, anti-inflammatory, antiviral, antioxidant and anti-tumor functions (15). In terms of antioxidant activity, Ebrahimi et al. (16) found that the antioxidant activity of pheophytin *b* was the highest, and the main structure determining their antioxidant effect was the aldehyde group. Further studies have shown that the antioxidant activity of Phide is due to its own ability to chelate ions and the stabilizing effect of porphyrin rings on reactive oxygen species (17). Phide is only produced naturally as a byproduct of Chl breakdown, making separation difficult and yield low. Chl is gradually destroyed during leaf aging, and Phide is only one of the transition stages and does not accumulate in considerable amounts, according to previous studies (18, 19). It is very difficult to extract Phide directly from nature. Most traditional extraction methods use organic solvents such as acetone, methanol, etc. (20), and it is difficult to avoid the residue of organic reagents in the extract, which has low safety. Some researchers have used acid hydrolysis and purification by thin-layer chromatography (21) and hydrolyzed Chl *a* with 30% hydrochloric acid, which only extracted Phide *a* with a low utilization rate of raw materials. Therefore, a safe, easy, and high-yield process for Phide synthesis is necessary. Currently, the primary Phide detection methods include high-performance liquid chromatography, UV–visible spectroscopy, fluorescence spectroscopy, and mass spectroscopy (21). However, these methods are time-consuming and complex. Therefore, another objective of this study was to develop a simpler and more intuitive method for detecting Phide concentration.

While possessing superior water solubility, Phide shares several vulnerabilities with Chl, including limited photothermal stability and susceptibility to acid, oxygen, and enzymatic degradation. These inherent limitations represent significant barriers to its practical use in food processing. Previous studies have shown that Chl can self-aggregate in solution to form aggregates that are more stable than monomers (22), and increasing concentration helps to enhance this self-aggregation effect. In high concentration state, the distance between Chl molecules is shortened, the weak interaction between Chl molecules is promoted, and the retention rate of Chl at high concentration is significantly improved compared to the dilute (23). The removal of the bulky phytyl chain reduces steric hindrance, potentially allowing Phide molecules to adopt a more ordered and compact spatial arrangement at high concentrations. This structural regularity is hypothesized to facilitate strong intermolecular interactions that counteract its inherent photo-instability. Previous studies have confirmed that charge transfer (CT) on the Chl porphyrin ring is a key link in the process of photosynthesis (24), and the study by Li et al. (23) also confirmed that CT is also one of the functions that cause the aggregation of Chl molecules. Compared with Chl, Phide has a more regular structure, and the contact rate between porphyrin rings is greatly enhanced by the removal of phytyl chain. Therefore, we hypothesize that aggregation may further enhance the CT between porphyrin rings, which is more conducive to improving its stability.

To harness Phide as a viable water-soluble colorant, this study constructs a comprehensive framework from macro-level characterization to micro-level mechanisms. Firstly, we synthesized and structurally characterized Phide, developing a streamlined HPLC-UV quantification method to accurately track its concentration. Utilizing this precise analytical tool, we investigated the critical bottleneck of Phide’s photostability, discovering that high-concentration treatment induces protective molecular aggregation. Finally, by employing quantum chemistry modeling, we elucidated the non-covalent interactions and charge transfer (CT) dynamics governing this aggregation. Unlike traditional synthetic dyes that incorporate metal ions, this study focuses on natural pigment derivatives, elucidating the aggregation mechanism of Phide molecules and proposing a method to enhance photostability without reliance on external conditions. Overall, these findings provide a robust mechanistic framework for the utilization of Phide in aqueous systems and facilitate the transition toward stable, clean-label natural pigments in the food, medical, and various other industries.

## 2 Materials and methods

### 2.1 Phide preparation

Phide was prepared from Chl as raw material and Chl is extracted from Spirulina (supplied by Hainan Xin Daze Biotechnology Co., Ltd.). The extraction was based on the method of Johnston with slightly modified (25). Since the Chl in Spirulina is Chl *a*, the Phide involved in this study is all Phide *a*. The sample was homogenized for 5 min by incorporating 200 mL of anhydrous ethanol and 400 mL of petroleum ether into 200 g of Spirulina powder. The homogenized material underwent centrifugation at 4 °C for 10 min at 8000 × g, followed by filtration and extraction of the supernatant. The separator funnel was filled with an equivalent volume of water to agitate and stabilize the separator, filtration, extraction and separation funnel. The upper oil phase (petroleum ether layer) was preserved, and an equivalent volume of water was employed for washing twice. The lower aqueous phase (the combined layer of ethanol and water) was extracted. The hydrocarbon phase was obtained, desiccated with anhydrous sodium sulfate, and subjected to filtration. The sample was placed in a rotary evaporator (N-1210BV-W, Ailang, Shanghai), which was maintained at 36 °C until around 200 mL of crude Chl extract was left. Petroleum ether-acetone (v:v = 9:1), petroleum ether-acetone (v:v = 7:3), and n-butanol-ethanol-water (v:v:v = 3:1:1) were utilized as eluents to extract carotene, lutein, and chlorophyll. The concentrated Chl was acquired with a purity of 86.6% following the collection of the Chl eluent.

The preparation of Phide was slightly modified based on the preparation of SCC (26, 27). Following the dissolution of the concentrated Chl solution in a 1:1 ethanol-water mixture, the pH was modified to around 10 using NaOH solution. The mixture was then heated and agitated in a water bath at 85 °C for 1.5 h, subsequently withdrawn and allowed to cool to room temperature. The extraction was conducted using petroleum ether. The petroleum ether phase of the upper layer became nearly colorless following many extractions. The aqueous Phide solution was produced by centrifuging and condensing the lower aqueous phase at 50 °C.

### 2.2 Apparent water solubility

After individually adding Chl and Phide to both the petroleum ether/water and pure water systems, thoroughly mixing, and allowing them to settle, their distribution and dissolution patterns (including the Tyndall phenomenon observed under light) were documented. Subsequently, both samples were centrifuged at 6000 × g for 10 min to check for precipitation.

### 2.3 Measurement of three-phase contact point

As outlined by Raziyeh Akbari and adapted by this study (28), the contact angle (*θ*) of Chl and Phide were determined at 25 °C utilizing a contact angle analyzer (OCA 25 AMP, Data physics Instruments, Germany). The sample was concentrated and dried on a square plastic sheet in a sequential manner. A precise volume of water (1.5 μL) was dispensed from a high-precision syringe and deposited on the surface of the sample. Subsequently, the droplet image was captured and digitized with an IDS camera attached to the instrument.

### 2.4 Spectrometric determination

The UV–Vis spectrum was determined using a microplate reader (Cytation5M, BioTek, United States) with full wavelength scanning in the wavelength range of 300–700 nm. The experiment was performed under light avoidance conditions. The fluorescence spectrum was measured using a microplate reader (Cytation5M, BioTek, United States) in accordance with the methodology outlined by Merzlyak et al. (29), utilizing a 96-well plate under room temperature conditions. The excitation wavelength was set to Ex = 446 nm, while the emission wavelength range was set to Em = 500–700 nm.

### 2.5 FTIR spectroscopy

Sample A was obtained by dissolving Chl in a 50% ethanol solution, and Sample B was obtained by dissolving Phide in a 50% ethanol solution. In accordance with the methodology proposed by Schestkowa et al. (30), a Fourier transform infrared spectrometer (iS10, Nicolet, United Kingdom) was employed to detect the Fourier transform infrared spectrum of the sample, with slight modifications. The sample and KBr were combined in a ratio of approximately 1:100 (m:m) and tested in the form of a tablet. At room temperature, the particles were scanned within the spectral range of 4,000–400 cm^−1^. To prevent the KBr background from influencing the signal strength, the amount of samples and the proportion of samples and KBr were strictly controlled during the test, and the test parameters were consistent. This allowed for the data to be quantitatively analyzed to the greatest extent possible.

### 2.6 Determination of Phide

#### 2.6.1 Determination of Phide concentration in HPLC

The SCC standard was obtained from Sigma-Aldrich. A sample of the SCC was accurately weighed to 9.87 mg and subsequently diluted to 100 mL with a mixture of acetonitrile and water (v:v = 80:20) to prepare the standard solution. For the sample solution, 2 mL of an aqueous solution of Phide was mixed with 8 mL of acetonitrile, and the mixture was thoroughly homogenized. The determinations were conducted using an Agilent liquid chromatograph equipped with a COSMOSIL Packed Column 5C18-AR (4.6 mm × 250 mm). The mobile phase consisted of acetonitrile and water (volume ratio 80:20) at a flow rate of 0.3 mL/min. The injection volume was set to 2.0 μL, and the column temperature was maintained at 30 °C. The detection wavelength was set at 405 nm.

The concentration of Phide was determined using SCC as an internal standard. The aqueous solutions of Phide were added to each sample group in volumes of 0, 40, 80, 120, 160, and 200 μL, respectively. Subsequently, 0.5 mL of the SCC standard solution was diluted to a total volume of 1.5 mL with a mixture of acetonitrile and water (v:v = 80:20). The samples were recorded as groups a, b, c, d, e and f for liquid chromatographic determination.

#### 2.6.2 Drawing of Phide concentration standard curve

The UV–Vis spectrum of Phide was determined using a microplate reader (Cytation5M, BioTek, United States) with full wavelength scanning in the wavelength range of 300–700 nm, and the experiment was performed under light avoidance conditions.

UV–Vis spectrum of Phide aqueous solutions at concentrations of 2.5 mg/L, 5 mg/L, 7.5 mg/L, 10 mg/L and 12.5 mg/L were determined and standard curves were plotted using Origin.

### 2.7 Preparation of samples with high concentration induced aggregation

Untreated Phide solution (17.1 mg/mL) served as the baseline control (sample A). The high-concentration Phide group (171 mg/mL) was used as experimental (sample B).

### 2.8 Determination of excitation wavelength

Firstly, 393 nm is selected as the initial excitation wavelength A_0_ (23). In order to determine another excitation wavelength, *A*_0_ is first used as the excitation wavelength to detect the emission spectrum, and *A*_m_, the position of the emission peak at this time, is selected as the excitation wavelength to detect the emission spectrum again, and the corresponding emission peak position *A*_n_ is recorded. Then *A*_n_ is used as the excitation wavelength, the position of the emission peak is detected and re-recorded as *A*_m_. This step is repeated until the excitation wavelength *A*_m_ almost overlaps the emission peak *A*_n_, then the wavelength *A*_m_ can be detected as the new excitation wavelength. It is verified by experiments that *A*_m_ = 330 nm.

### 2.9 Quantum chemical calculations and wave function analysis

The geometric optimization, vibration analysis and excited state calculation of the molecular structure were all implemented by Gaussian 16 (A.03). In this study, quantum chemical calculations were performed using density functional theory (DFT) and time-dependent density functional theory (TD-DFT) methods. The geometric optimization was realized by B3LYP function combined with 6-31G (d) base group (31). The optimized structure had no virtual frequency. The excited state was calculated using the CAM-B3LYP-D3 (BJ) function combined with the 6-31G (d) base set (32). Molecular color prediction, electrostatic potential (ESP), van der Waals (vdW) potential, non-covalent interaction (NCI), independent gradient model (IGM) analysis, and hole–electron analysis were performed using Multiwfn 3.8 (33). Visual Molecular Dynamics (VMD) software was used to map the molecular structure model and its isosurface.

### 2.10 Data processing and statistical analysis

All data were expressed as mean ± standard deviation, and each experiment was repeated at least three times. The analysis of variance was conducted using SPSS 22.0 software, and the Duncan multiple range test was employed to analyze the experimental data. A *p*-value of less than 0.05 was considered statistically significant (in the data presented in the chart below, the difference between superscript letters indicated a statistically significant difference).

## 3 Results and discussion

### 3.1 Water solubility between Phide and Chl

**Figure 1:**
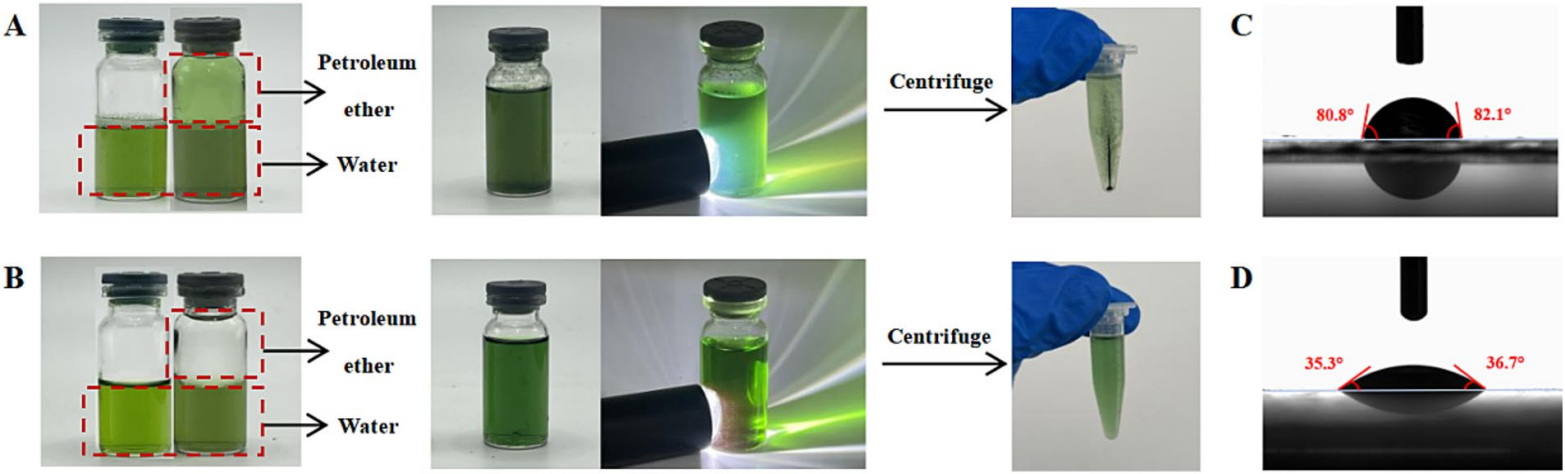
Comparison of the water solubility of Chl and Phide in water: Apparent water solubility of Chl **(A)** and Phide **(B)**; three-phase contact point of Chl **(C)** and Phide **(D)**.

Chl and Phide display distinct solubility characteristics in aqueous solutions (Figures 1A,B). Phide surpassed Chl in aqueous solubility. Figure 1A illustrated Chl were simultaneously dissolved in water/petroleum ether system. Due to its fat-solubility, Chl was more effectively dissolved by organic reagents, a portion of Chl dissolved in the petroleum ether. Owing toalgae possess natural Chl derivatives devoid of a phytyl chain (34, 35), aqueous solution appears green. Moreover, a portion of Chl dissolved in the aqueous phase. The residual Chl dissolved in water, forming a turbid solution. Figure 1B illustrated Phide were simultaneously dissolved in water/petroleum ether system. Phide demonstrated complete solubility in aqueous media, while the petroleum ether phase retained its colorless and transparent characteristics, confirming the hydrophilic nature of Phide. Under photostimulation, the aqueous solution of Chl developed turbidity accompanied by Tyndall scattering phenomena, indicating the presence of colloidal particles. Subsequent centrifugation resulted in visible precipitation of Chl. In contrast, Phide maintained a homogeneous colloidal dispersion in aqueous solution with no detectable precipitate formation post-centrifugation. These observations indicate distinct solubility behaviors between Phide and Chl. The characteristic red fluorescence of Phide solution is emitted under ultraviolet light. The fluorescence of Phide was red, and the reactive group was the porphyrin ring. This fluorescence was characteristic of Chl and its structural analogs (36). After absorbing photon energy, Chl molecules in the ground state transition to a highly excited state and emit fluorescence as they de-excite. Figures 1C,D illustrated the three-phase contact point (*θ*) of Chl and Phide. The *θ* of a substance can be used to reflect its hydrophilic and hydrophobic properties. A larger *θ* indicates a more hydrophobic sample, while a smaller *θ* indicates a more hydrophilic sample (37). The *θ* of Chl and Phide were found to be 81.45 ± 0.65° and 36 ± 0.7°, respectively. A comparison of the θ values demonstrates that Chl exhibited a pronounced hydrophobic character. In contrast, Phide displayed a marked hydrophilic character, indicating excellent water solubility. The apparent phenomena of water solubility of the two (Figures 1A,B) and *θ* (Figures 1C,D) demonstrated consistent results, thereby confirming that Phide has excellent water solubility.

### 3.2 Spectroscopic properties of Phide and Chl

**Figure 2:**
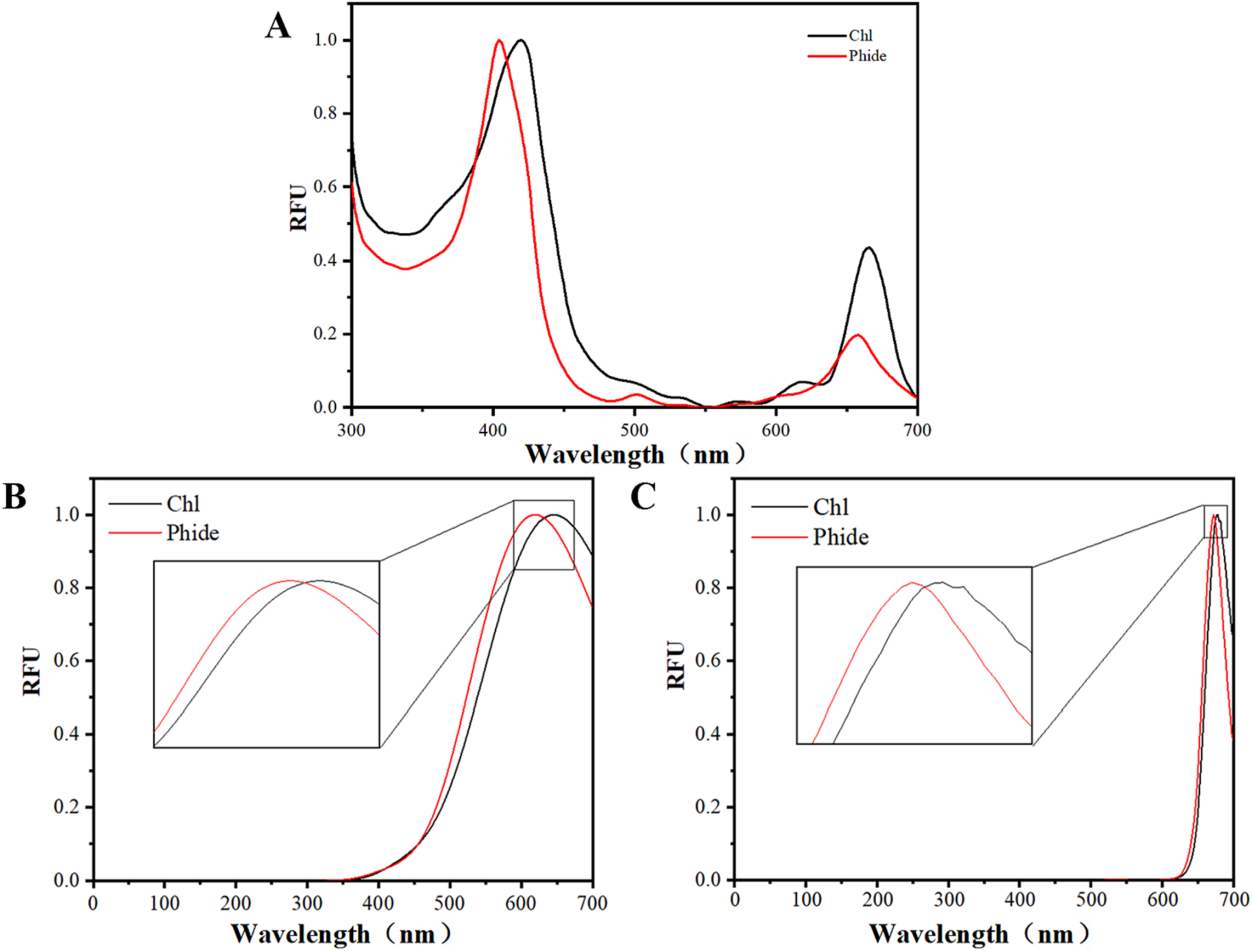
Spectral properties of Chl and Phide: UV–Vis absorption spectrum **(A)**; fluorescence spectra calculated by quantum chemistry **(B)**; fluorescence spectra of actual measurements **(C)**.

The central Mg ion situated at the core of the macroring was initially eliminated under acidic conditions, substituted by two hydrogen ions, and transformed into pheophytin. Subsequently, Phide was generated through a deester reaction on the phytyl chain (38). Consequently, Phide represented the Chl porphyrin ring structure devoid of the central Mg ion and phytyl chain. Porphyrins exhibited two UV–Vis absorption bands: the Soret band and the Q band. The Soret band, ranging from 400 to 500 nm, was a significant absorption band. The *π*-π* transition involving 18 electrons from the macrocycle in the porphin ring generated a delocalized π bond, while the π-π* transition produced the Q band inside the 450 to 700 nm range (39). The maximum absorption wavelength of the porphyrin absorption spectrum was contingent upon the outer substituents of the ring. Figure 2A demonstrated that the Soret band absorption peak of Phe shifted from 420 nm to 404 nm in comparison to Chl. Porphyrin molecules form hydrogen bonds when hydrogen atoms substituted the *β* site of the ring, resulting in a blue shift of the Soret band. This confirmed that hydrogen atoms replaced the phytyl chain. Phide exhibited a blue shift and attenuation at all four Q band peaks. Intense absorption peaks at 500 and 660 nm may resulted from the more ordered configuration of porphyrin ring molecules following the substitution of hydrogen atoms for the phytyl chain. This diminished steric hindrance, decreased the energy level of bonding orbitals, elevated the energy level of anti-bonding orbitals, and increased the energy gap between the highest occupied and lowest vacant orbitals. The replacement of the center Mg ion of the porphyrin ring with two hydrogen ions resulted in a more symmetrical structure, which in turn gave rise to a simplified Q-band pattern (40). Consequently, the absorption peak of Phide in the Q-band region was weakened.

With regard to their fluorescence properties, porphyrins employ the Soret band or Q band as the excitation wavelength, both of which can elicit fluorescence emission of varying intensities at wavelengths between 600 and 750 nm (39). Quantum chemical computations can determine the fluorescence spectra of Chl and phenol in water. The distinct chemical structures of Chl and Phide resulted in variations in excitation peak positions. Figure 2B illustrated the theoretical fluorescence spectra in aqueous solution, which have been calculated using quantum chemistry. The excitation peak of Phide was observed at 620 nm, while that of Chl was seen at 645 nm. The excitation peak of Phide exhibited a blue shift relative to Chl. The fluorescence spectra, as measured, were presented in Figure 2C. Upon excitation at 446 nm, the emission peaks of Phide and Chl were observed at 672 nm and 678 nm, respectively, and the blue shift effect was also evident at this time. The changes in the actual molecular structure demonstrated a consistent trend with the quantum chemical simulation.

### 3.3 FT-IR spectrum

**Figure 3:**
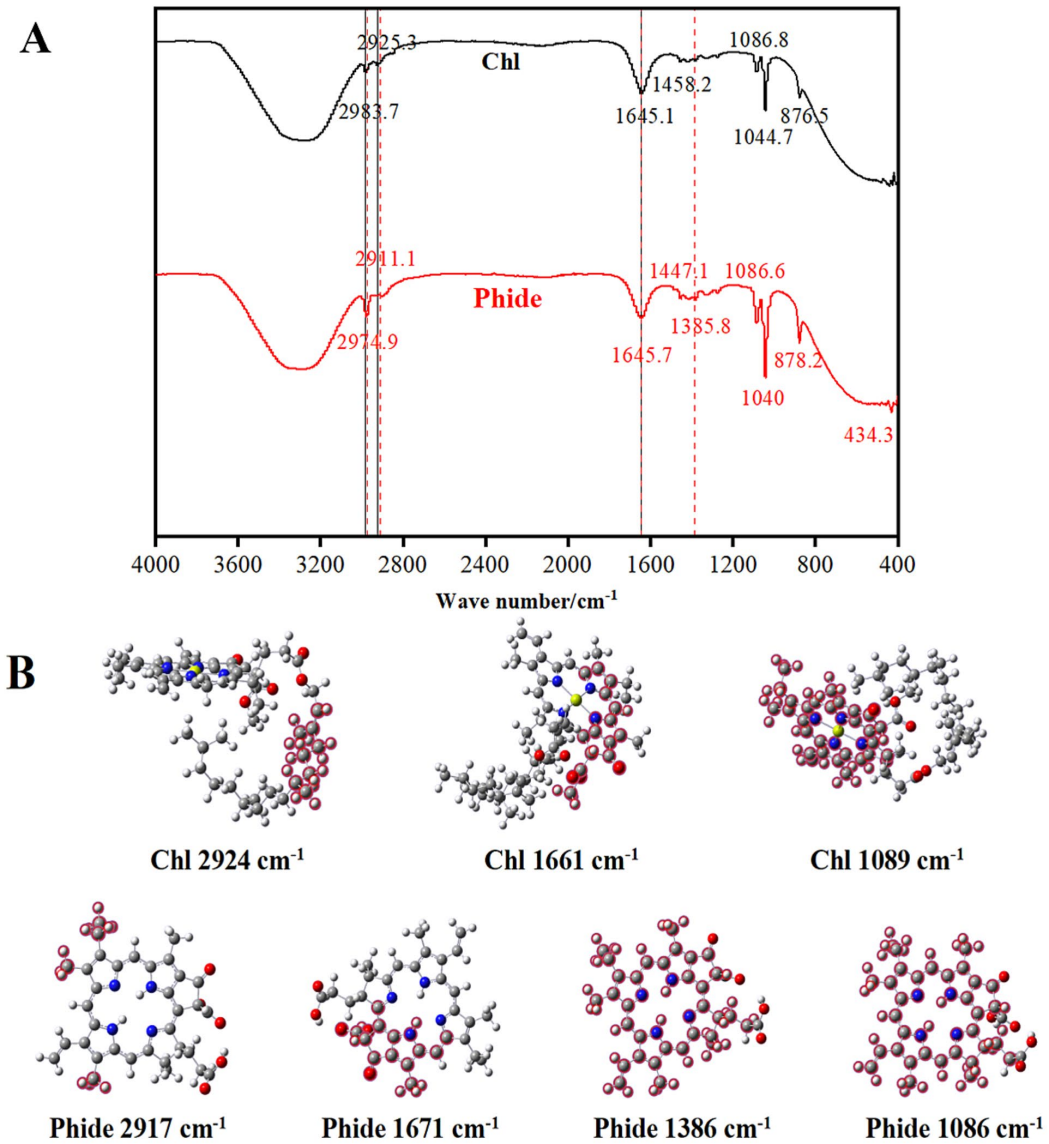
FT-IR spectrum **(A)** and molecular vibration schematic **(B)** of Chl and Phide.

Figure 3 illustrated the FTIR spectra of Chl and Phide (A) and the results of quantum chemical simulations of molecular vibrations (B). The atoms highlighted in red were those that produce significant vibrations at corresponding wave numbers. The stretching vibration of the saturated carbon C-H bond was observed at 3000–2700 cm^−1^. Chl exhibited two peaks within this range, at 2983.7 cm^−1^ and 2925.3 cm^−1^. Nonetheless, Phide’s two peaks shifted to 2974.9 cm^−1^ and 2911.1 cm^−1^. The quantum chemistry molecular vibration diagram indicated that Chl exhibited vibrations at 2984.51 cm^−1^ and 2924.72 cm^−1^, attributed to the stretching vibrations of the C-H bond in the phytyl chain, whereas Phide vibrated at 2975.08 cm^−1^ and 2917.60 cm^−1^, corresponding to the porphyrin ring. The stretching vibrations of double bonds such as C=C and C=N, along with the skeletal vibrations of the porphyrin ring, resulted in the absorption peak at 1690–1500 cm^−1^. Within this range, Chl absorption reached a maximum at 1645.1 cm^−1^, while Phide peaked at 1645.7 cm^−1^. The quantum chemical molecular vibration diagram indicated that the vibrations of Chl and Phide at 1661.94 cm^−1^ and 1671.10 cm^−1^, respectively, were attributed to the C=C double bond of the porphyrin ring, confirming the integrity of their structures. A novel Phide absorption peak was detected at 1385.8 cm^−1^. The quantum chemistry schematic diagram of molecular vibration indicated that Phide’s vibration occurred at 1386.32 cm^−1^. This vibration originated from the majority of porphyrin ring atoms. Quantum chemistry computed the vibrational frequencies of Chl and Phide at 1089.73 cm^−1^ and 1086.6 cm^−1^, respectively. Both vibrations originate were from the majority of porphyrin ring atoms. This indicated that in Phide, the expansion vibration of atoms originally located on the side chain of Chl plant groups disappears, indicating that the phytyl chain structure in Phide no longer exists, while the expansion vibration of atoms originally located on the porphyrin link points of Chl still exists, indicating that the porphyrin structure is intact.

### 3.4 Theoretical color estimation of Chl and Phide based on calculated UV–vis spectra

**Figure 4:**
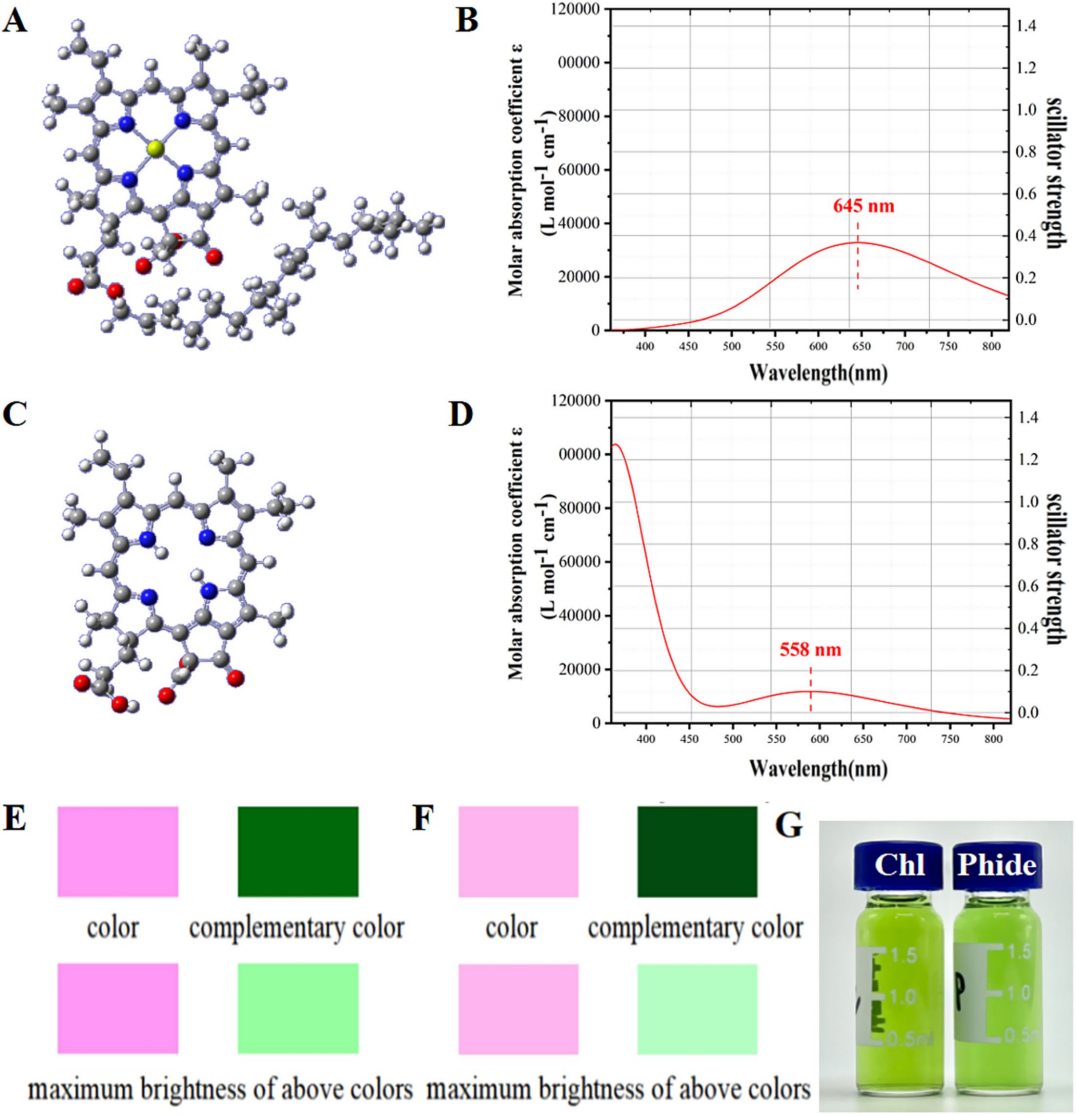
Color of Chl and Phide: molecular structure of Chl **(A)** and Phide **(C)**; UV–Vis absorption spectra calculated by quantum chemistry of Chl **(B)** and Phide **(D)**; schematic representation of the estimated color based on calculated spectra of Chl **(E)** and Phide **(F)**; the actual color of Chl in ethyl alcohol and Phide in water **(G)**.

Chl and Phide molecules were depicted in Figures 4A,C. The modeling of Chl dissolution in ethanol and Phide dissolution in aqueous solution took into account their unique solubility characteristics. The UV–Vis spectrum of the substance can be calculated by quantum chemistry, and the absorption and reflection colors of the substance can be obtained according to the position of the absorption peak of the spectrum. Figures 4B,D illustrated the calculated UV–Vis spectra of Chl in an ethanol solution and Phide in an aqueous solution. In the visible range, the absorption peak of Chl was observed at 645 nm, while the absorption peak of Phide was observed at 558 nm. Figures 4E,F depicted the color of the Chl and Phide spectra, the complementary color, and these colors at maximum brightness. This resulted in spectral and complementary colors exhibiting only chromatic variations without any differences in brightness. Absorption spectra derived from material color prediction typically favor the lower right quadrant. Chl exhibited a lighter hue and inferior color effect in ethanol solution compared to Phide, which was darker and demonstrated a superior color effect in water. Consequently, an alternative Chl spectroscopic detection method may be formulated. Upon establishing the detection method in 3.5, Figure 4G illustrated the coloration of Chl and Phide at identical concentrations in solution. Phide exhibited a deeper green hue and superior color fidelity, indicating that the structural changes predicted by theory matched the actual structural changes.

### 3.5 Determination method of Phide concentration

**Figure 5:**
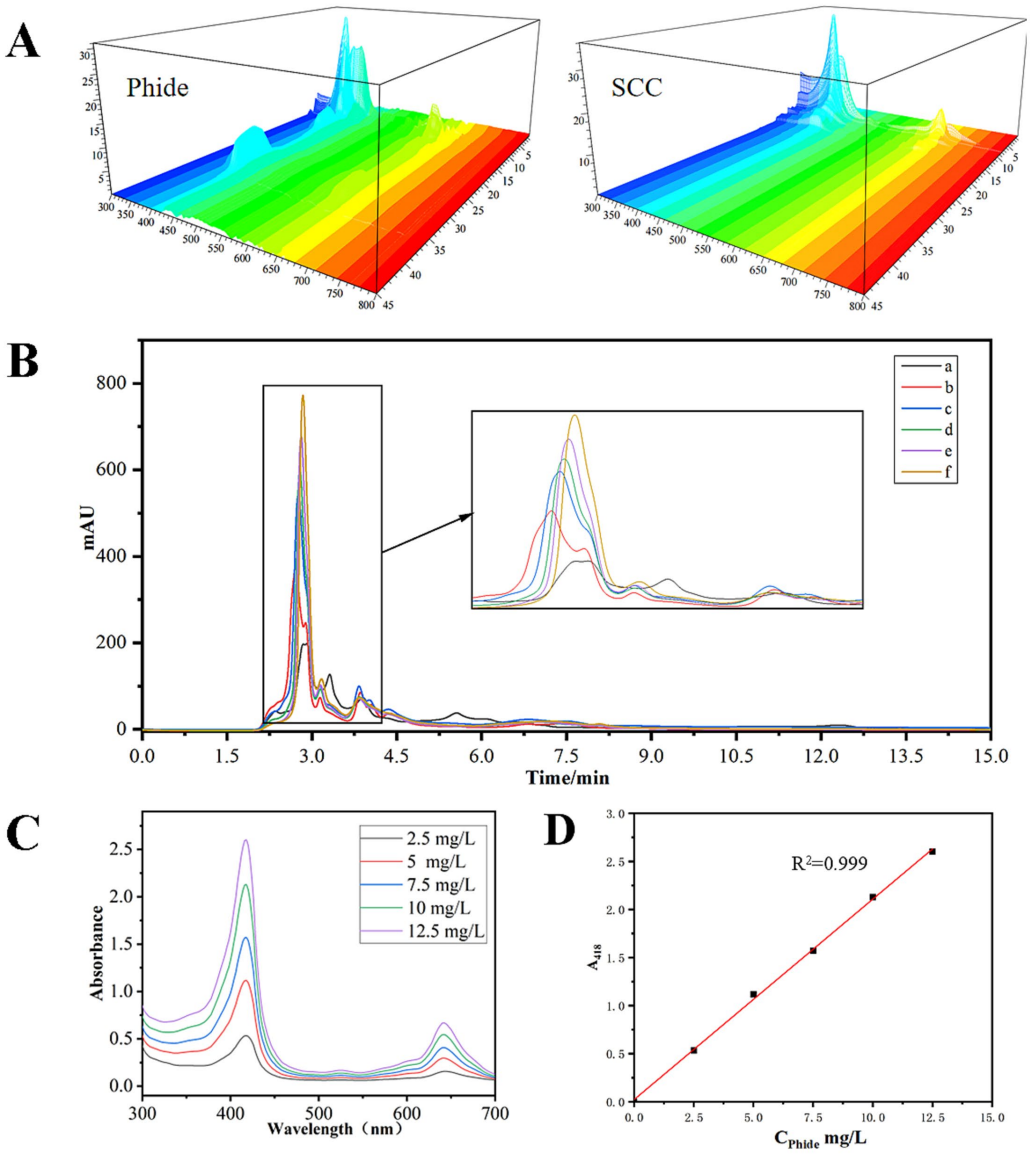
Determination of Phide concentration: HPLC-DAD spectrum of Phide and SCC **(A)**; HPLC of Phide and SCC **(B)** (amount of Phide aqueous solution added: a: 0 μL; b: 200 μL; c: 400 μL; d: 600 μL; e: 800 μL; f: 1000 μL); UV–VIS absorption spectrum of different concentrations of Phide **(C)**; standard curve of concentration and absorbance of Phide **(D)**.

Figure 5A illustrated the HPLC-DAD profile of Phide alongside the internal standard substance SCC. The appearance of characteristic peaks for both Phide and SCC was observed after a period of 5 min. Despite the retention time of these characteristic peaks was different, the UV–VIS spectra corresponding to these peaks exhibited consistent absorption characteristics, indicative of a porphyrin structure. This was illustrated in Figure 2A. Both Phide and SCC were gradually eluted. This may be attributed to the aggregation of Phide and SCC in the solvent, which exhibited disparate hydrophobic characteristics on the aggregate surface contingent on the degree of aggregation. The aggregates were distinct from free Chl and Phide and were retained within the chromatographic column. They were subsequently dispersed into monomers under the gradual elution of the eluent, resulting in their gradual release from the chromatographic column (23). In this experiment, the Phide was prepared from Chl with a purity of 86.6%, the SCC standard solution was composed of SCC standard products, and the system was devoid of compounds other than porphyrins. It is therefore hypothesized that the characteristic peaks obtained by HPLC from Phide and SCC at different times originated from the same substance. Consequently, the sum of all characteristic peak areas will be employed to calculate the concentration in subsequent experiments. Figure 5B illustrated the HPLC chromatogram of a mixed solution containing varying concentrations of Phide alongside a constant SCC concentration. Variations in Phide concentrations within a system with a constant SCC content influenced the characteristic peak area. The integrated areas of all characteristic peaks in the Phide HPLC chromatograms at varying concentrations and a constant SCC concentration were computed. The aggregate area of the characteristic peak (*y*) exhibited a linear correlation with Phide concentration [*x*(mg/L)] in the groups, represented by the standard curve *y* = 2002.8*x* + 4612.7 and a *R*^2^ value of 0.9361. Accordingly, the concentration of Phide and SCC in the mixed solution could be calculated on the basis of the relationship between the total area of the characteristic peaks in the HPLC chromatogram. The concentration of the Phide concentrated solution could be calculated on the basis of the ratio of the amount of substance. The calibration relationship used for quantitative analysis is presented in Equation 1, whereas the concentration conversion and retention rate calculations are described in Equations 2, 3 respectively. Table 1 presented the calculations for groups a and f.


$$\begin{array}{l} C_{\text{Phide}}\;(\mathrm{mg}/L)=[(15779-5438.6)/5438.6]\\ {}*0.04935^{*}578.66/\\ (724.15^{*}0.0002)=374.9 \end{array}$$ (1)

**Table 1:** Phide concentration calculation table.

Group	Total volume of solution (mL)	SCC standard solution addition amount(mL)	SCC content (mg)	Amount of Phide aqueous solution added(mL)	Phide content (mg)	Total peak area
a	1.5	0.5	0.04935	0	0	5438.6
f	1.5	0.5	0.04935	0.2	0.07498	15,779

In this instance, Mr_Phide_ = 578.66, Mr_SCC_ = 724.15.

The UV–Vis spectrum was obtained following the gradient dilution of the concentrated Phidephorbide aqueous solution. Figure 5C illustrated that the porphyrin ring, the principal structure of Phide, exhibited increased absorption at a specific wavelength within the Soret band (400–500 nm) as the solution concentration rose. Upon examining the linear relationship between the concentration of Phide aqueous solution [y(mg/L)] and absorption values (x) across different wavelengths, the most robust correlation was identified at 418 nm. The standard curve depicted in Figure 5D was represented by the equation:


$$y=0.0261x+0.022,R^{2}=0.999$$ (2)

Phide concentration exhibited a strong correlation with UV–Vis spectrum absorption at 418 nm, referred to as *A*_418_.

The quantitative assessment of Phide concentration in the *A*_418_ system was precise. The solution at 374.9 mg/L was diluted 40 times to ascertain its *A*_418_ value, which measured 1.983. Consequently, a formula for Phide concentration was developed.


$$C_{\text{Phide}}\;(\mathrm{mg}/L)=4.726^{*}\;A_{418}$$ (3)

The method was subjected to repeated testing, yielding an RSD of 0.65%. In this study, the method validation was primarily focused on the aqueous model system to support the mechanistic exploration of aggregation-induced stability. While the current results demonstrate high linearity and specificity, future applications in complex food matrices may require additional validation steps, such as matrix effect evaluation and recovery testing, to adapt the protocol for specific commercial products.

### 3.6 High concentration aggregation improves the stability of Phide

**Figure 6:**
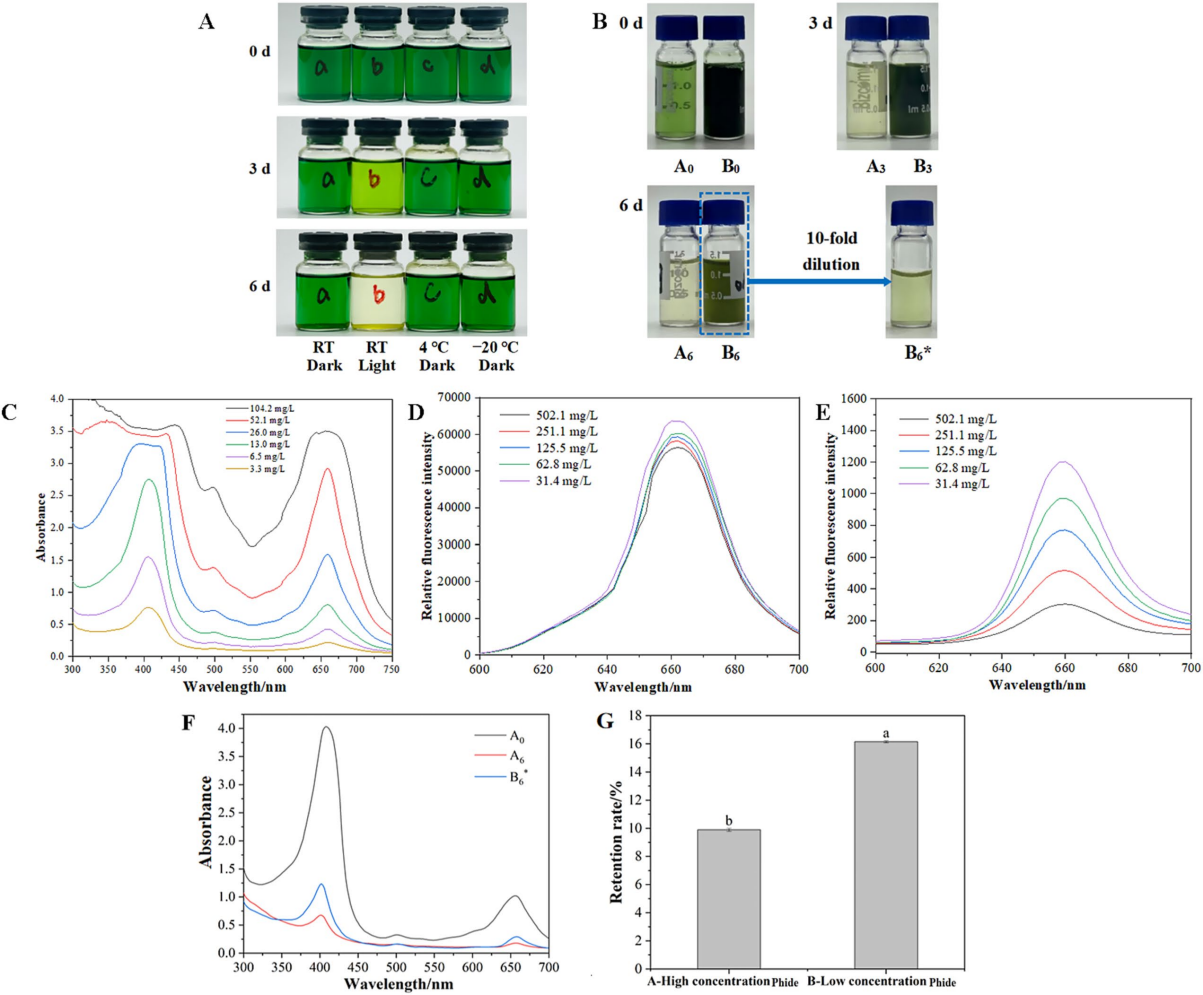
Aggregation induced by high concentration enhances Phide stability: influence of common food storage conditions on color stability **(A)**; color change of high concentration Phide (sample A) and low concentration Phide (sample B) **(B)**; UV–Vis spectra **(F)**; retention rate of Phide **(G)** after 6 d in normal light, RT; UV–Vis absorption spectrum of Phide **(C)**; fluorescence emission spectra of Phide solution at 330 nm **(D)** and 393 nm **(E)**.

The degradation under four typical food storage conditions was simulated in Figure 6A. The Phide samples exhibiting the greatest discoloration were maintained at room temperature and exposed to light. An analysis of the four sample groups indicated that illumination was the primary factor influencing Phide discoloration, whereas temperature exerted minimal impact. This corroborates earlier findings regarding Chl discoloration and temperature (22). Prior research had demonstrated that Chl possesses the capacity for self-aggregation, whereby an increase in Chl concentration within a system can facilitate the self-aggregation of Chl molecules, thereby enhancing the retention rate (23). Given that Phide exhibits a more orderly structure compared to Chl, increasing Phide concentration and reducing intermolecular distance can foster a more organized arrangement, bolster intermolecular interaction, and refine self-aggregation. This aggregation may facilitate intermolecular interactions, including possible charge transfer effects.

As demonstrated in Figure 6C, the UV–VIS absorption spectra of Phide aqueous solutions with varying concentrations are presented. At varying concentrations, the positions of the characteristic absorption peaks of each sample remained constant, indicating Soret band absorption at 400–450 nm and Q band absorption at 500 nm and 660 nm, which corresponded to the characteristic peaks of porphyrin rings in the Phide structure. This observation suggests that the system does not undergo any new substance formation, and that the alterations in its properties can only be attributed to the aggregation of Phide itself. As illustrated in Figures 6D,E, the fluorescence emission spectra of Phide solution were measured with 330 nm and 393 nm as excitation wavelengths, with a concomitant decrease in concentration from 502.1 mg/L to 31.4 mg/L. At 330 nm, the fluorescence emission intensity is strong, and fluorescence quenching occurs in the solution with the increase of solution concentration. Conversely, at 393 nm, the fluorescence emission intensity is weak, yet with an increase in solution concentration, the phenomenon of fluorescence quenching is also observed. The results obtained at both 330 nm and 393 nm demonstrate a consistent pattern: namely, that the fluorescence intensity diminishes in proportion to the rise in solution concentration. This concentration-dependent fluorescence quenching is a direct macroscopic manifestation of aggregation-induced intermolecular interactions. Specifically, as molecules aggregate at high concentrations, non-radiative decay pathways are activated. The exact quantum mechanical origin of this quenching—intermolecular charge transfer (CT) effects—will be theoretically elucidated in Section 3.8.

Figure 6B illustrated Phide samples exhibiting a tenfold concentration disparity. Sample B6 exhibited superior color retention compared to sample A6 after 6 days of exposure to light at room temperature. Figure 6F illustrated the sample UV–Vis spectrum. Upon dilution of B6 to B6*, the UV–Vis spectra of A6, B6, and B6* exhibited Phide-like profiles, signifying that the structural configuration of Phide within the system remains unaltered. The retention rate of the Phide samples was illustrated in Figure 6G. Sample A showed 9.89 ± 0.10% retention after 6 days storage while sample B retained rate was 16.15 ± 0.07% under identical conditions (*p* < 0.05). Compared with sample A, the retention rate of sample B was improved by 63.3%. The 63.3% enhancement in retention indicates that high-concentration-induced aggregation may provide a relative stabilizing effect against light-induced degradation, although Phide remains sensitive to environmental factors.

### 3.7 Interaction analysis of Phide dimer in aqueous solution

**Figure 7:**
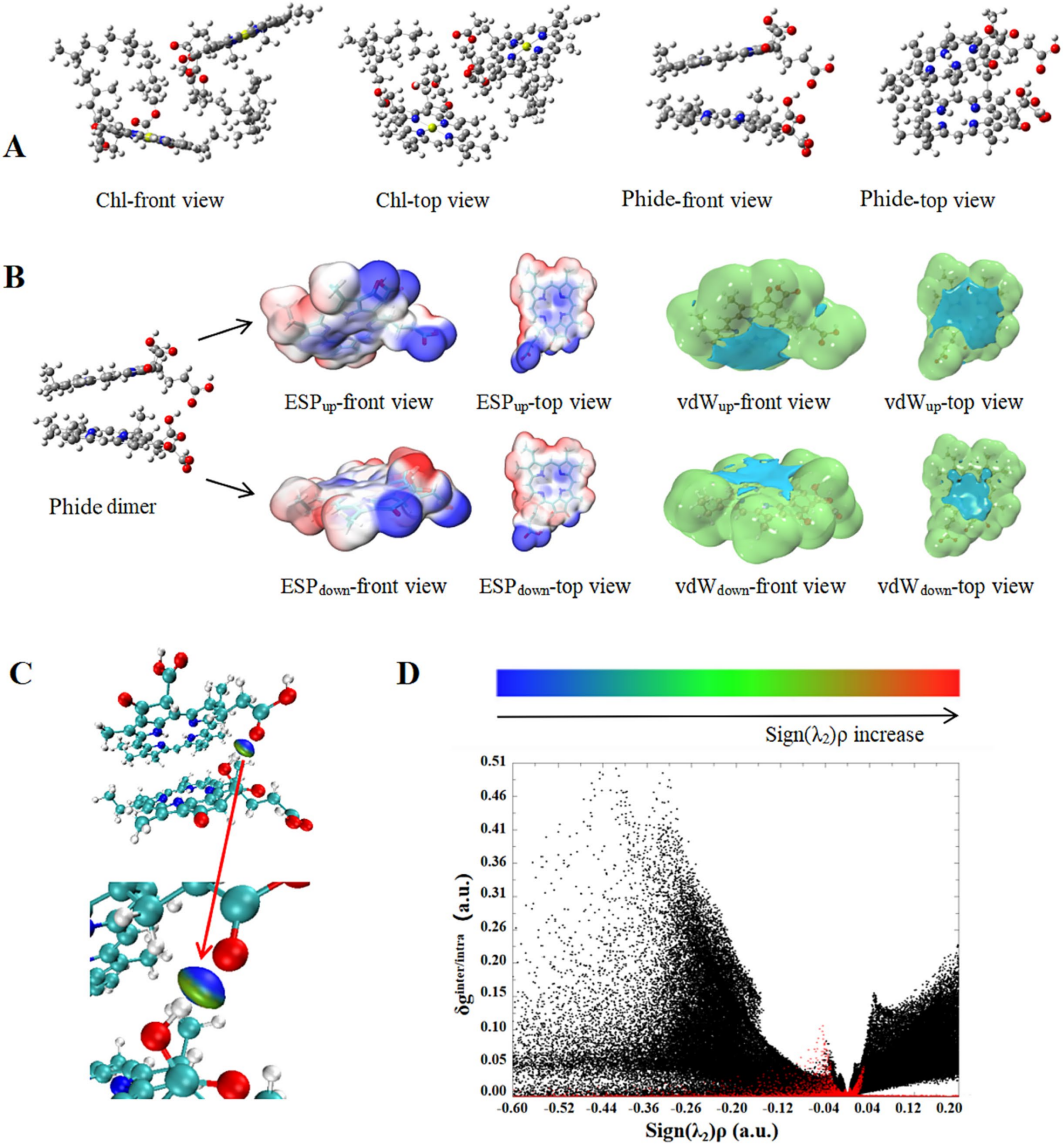
Intermolecular interactions of Phide dimer: dimer configuration of Chl and Phide **(A)**; ESP and vdW potential of Phide dimer in water **(B)**; IGM analysis of Phide dimer at Isovalue = 0.02 **(C)**; IGM scatter chart **(D)**.

The aforementioned phenomenon demonstrates that aggregation, induced by high concentration in aqueous solution, augments the stability of Phide. The self-aggregation in aqueous solution was believed to result from a reduction in intermolecular spacing and an enhancement of interaction forces. Dimer is the smallest aggregate unit and is an excellent model for studying the aggregation mechanism. The dimer was used here as a simplified theoretical model to represent possible intermolecular interactions within aggregated structures. The interaction force of the dimeric structure of Phide in aqueous solution was examined to ascertain whether the noted enhancement in stability was attributable to intermolecular self-aggregation. The stable dimer configuration of Chl and Phide in aqueous solution was simulated using quantum chemistry calculations, as depicted in Figure 7A. In aqueous solution, the two porphyrin rings of Chl were cross-oriented, with the phytyl chain of one molecule positioned adjacent to that of another. In contrast, the two porphyrin rings of Phide were oriented parallel to each other, with the oxygen-containing groups arranged in a stacked configuration on the side. In aqueous solution, the single point energies of Chl dimer and Phide dimer were lower than the cumulative single point energies of two Chl and Phide monomers (Table 2). The findings indicated that Chl and Phide underwent self-aggregation in aqueous solution. Furthermore, binding energy of Phide dimer possessed 2.68 times of Chl dimer in an aqueous solution, which indicated that Phide’s self-aggregation exhibited greater stability than that of Chl.

**Table 2:** Monomer energy, dimeric energy and binding energy of Chl and Phide.

Sample	Monomer energy (Hartree)	Dimeric energy (Hartree)	Binding energy (Hartree)
Chl	−2934.764713	−5869.533210	0.003784
Phide	−1911.339922	−3822.689975	0.010131

Phide dimers exhibited a stable configuration when stacked in parallel, which provided a structural basis for analyzing the interaction forces between molecules. The prevailing view was that the main sources of intermolecular interaction were electrostatic interaction, dispersion attraction, exchange repulsion, CT and polarization effect. Of these five interactions, the electrostatic interaction and dispersion have been the subject of considerable attention as the primary driving forces. In light of this, a further investigation was conducted into the electrostatic potential (ESP) and van der Waals (vdW) potential of Phide dimers, with a particular focus on their aggregation mechanism. Figure 7B illustrated the ESP and vdW potential of the Phide dimer. The ESP of the Phide dimer was observed, with red and blue indicating regions where ESP was positive and negative, respectively. A comparison of the ESP distribution on the two molecular oxygen-containing groups revealed that there was a notable negative ESP around the oxygen-containing group on the molecular side above the dimer, while there was a distinct positive ESP around the hydrogen atom on the molecular side chain below the dimer. Therefore, the oxygen-containing group exhibited a more pronounced interaction with the hydrogen atom when Phides form a dimer. Nevertheless, other positions within the molecule exhibited the same potential distribution when they were in close proximity, yet they can still be firmly combined, suggesting the presence of additional interacting forces beyond electrostatic forces. In the case of polar intermolecular interactions, the electrostatic action is the dominant force, with the vdW potential action playing a secondary role. Figure 7B illustrated the vdW potential isosurface of two molecules in a Phide dimer with Ne atom as the probe. The colors green and blue denoted the positive and negative isosurfaces, respectively. The negative region was significant, where the probe atom could attach to the molecule. The data indicates that the negative isosurface was located within the cavity region at the center of the porphyrin ring. This indicated that the probe atom, Ne, and several small molecules characterized by low polarity and the absence of pi hydrogen bonding exhibited a strong binding affinity within this region.

Figures 7C,D depicted the Phide dimer weak interaction IGM and scatter plot, which offered a more intuitive representation of the intermolecular binding sites. As sign(*λ*_2_)*ρ* increased, the intermolecular interaction underwent a change. Blue represented a strong electrostatic attractive force, green represented vdW action, and red represented a strong mutual repulsion. The red scatter in the IGM scatter diagram reflected the intermolecular interaction, while the black scatter reflected the intramolecular interaction. A combination of the IGM and scatter diagram revealed that the red scatter points were concentrated on both sides of sign(*λ*_2_)*ρ* = 0, with a greater distribution on the side of sign(*λ*_2_)*ρ* < 0. This indicated that the strong attraction between molecules is dominant in the Phide dimer, with the effect of vdW force being relatively weak. The strong attraction between the two Phide molecules was primarily manifested in the side chain between the oxygen-containing group and the hydrogen atom, namely the hydrogen bond, where it is more readily formed and contributes to the stability of the dimer structure. In light of these findings, the ESP analysis, vdW potential analysis, and IGM analysis had collectively demonstrated that the formation and structural stability of the Phide dimer was predominantly governed by the formation of hydrogen bonds, with vdW forces exerting a secondary influence.

### 3.8 CT in Chl *a* dimer in ground-excited state

**Figure 8:**
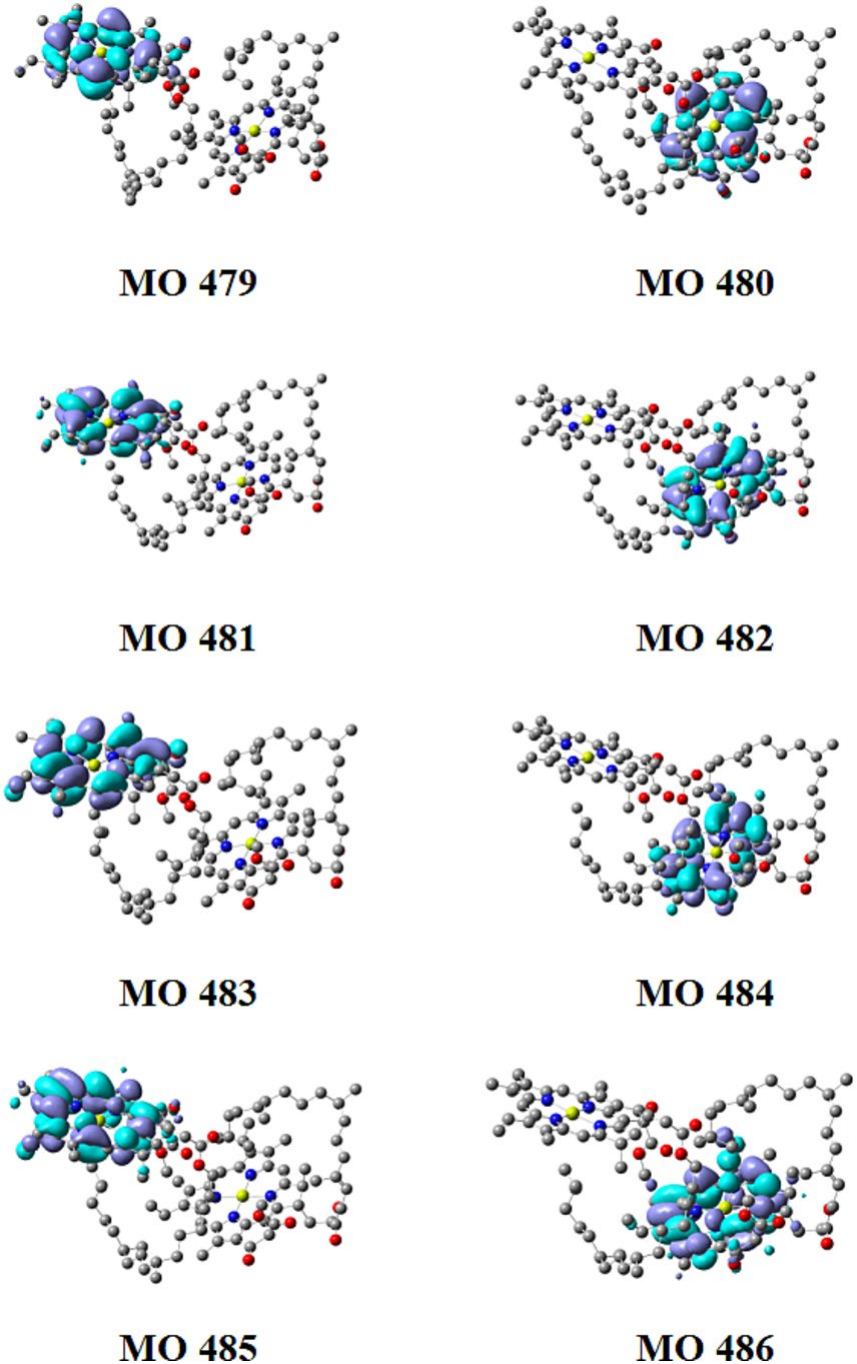
Diagram of tracks 479 to 486 from Chl.

**Figure 9:**
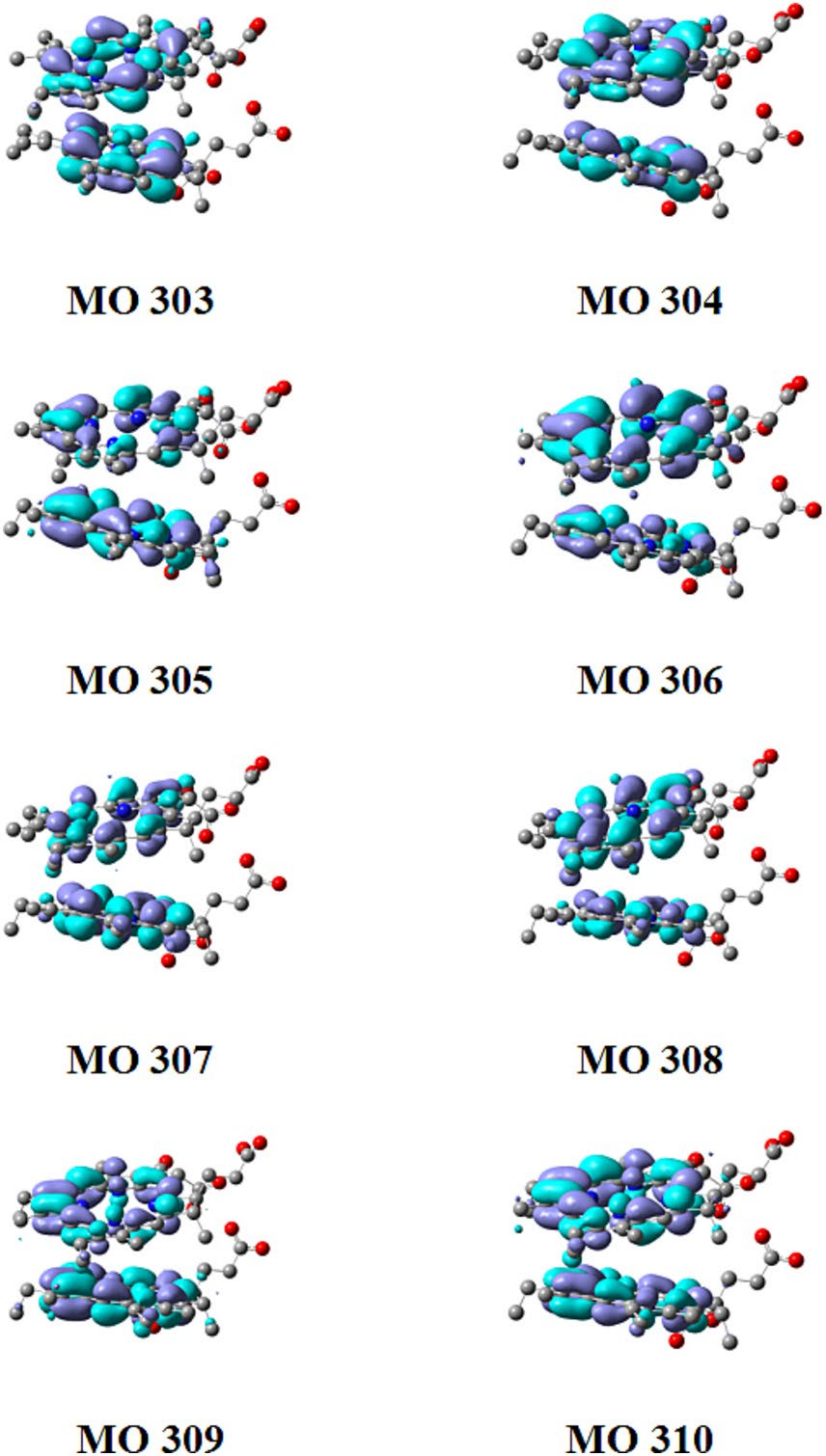
Diagram of tracks 303 to 310 from Phide.

**Figure 10:**
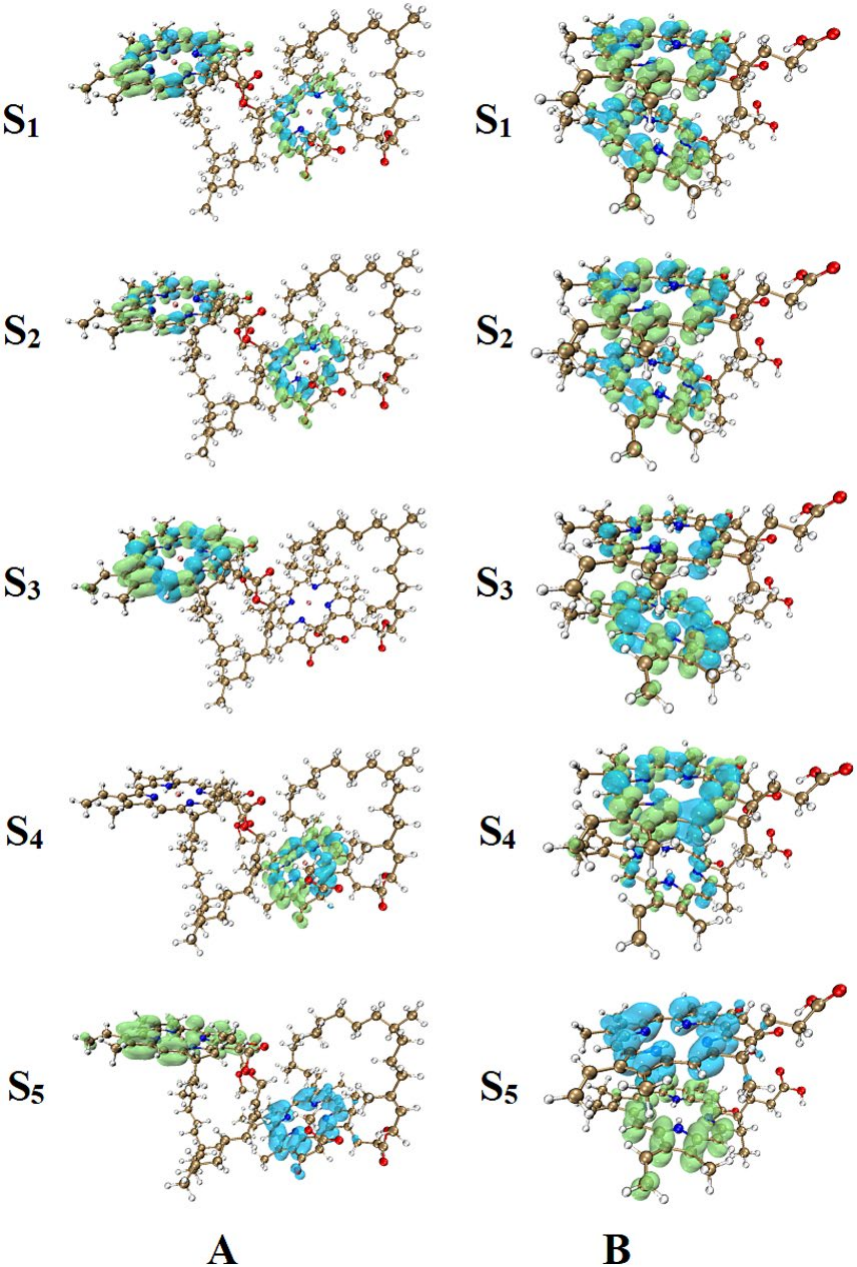
Hole–electron analysis of excited Chl dimer **(A)** and Phide dimer **(B)**.

Supplementary Tables S1, S2 showed the orbital contributions of the Chl dimer and Phide dimer in the five excited states from S_1_ to S_5_. Figures 8, 9 shown a schematic of its orbit. In Figure 10, the hole–electron analysis of the Chl dimer and Phide dimer in S_1_-S_5_ was shown these resembled the distribution region before and after electron excitation, or electron travel route. The blue region in Figure 10 represented the hole region in the excited state, where electrons leave, and the green region represented the excited state area, where electrons arrive. According to the orbital contribution rate of the Chl dimer in Figure 10A, the electron transition from orbitals 481–483 contributed the most to S_1_, 482–484 to S_2_, 479–483 to S_3_, and 480–484 to S_4_. Upon examining these results in conjunction with the hole–electron analysis of S_1_-S_4_, it became apparent that the regions of electron distribution in the excited states of S_1_-S_4_ were situated on the porphyrin rings of the two monomers in the dimer. Electron transfer did not transpire between the two monomers; instead, the electrons relocated within the same monomer, indicative of a typical local excitation. The largest contribution to S_5_ was the electron transition from orbitals 482–483. This precisely aligns with the HOMO-LUMO orbital transition. In conjunction with the hole–electron analysis of S_5_, it could be seen that the regions where electrons leave and the regions where electrons arrive were located on the two monomers in the dimer, respectively. This indicated that the hole and electron regions remaining completely separated. This was a representative example of a CT excitation, with an excitation energy of 3.357 eV. According to the orbital contribution rate of the Phide dimer in Figure 10B, the electron transition from orbitals 306–307 contributed the most to S_1_, which corresponds to the HOMO-LUMO orbital transition, 305–307 to S_2_, 303–307 to S_3_, and 304–308 to S_4_. The hole–electron analysis of S_1_–S_4_ indicated that the electrons was a hallmark of local excitation. The largest contribution to S_5_ is from the electron transition of orbitals 306–307. When combined with the hole–electron analysis of S_5_, it could be observed that was also a typical CT excitation, with the excitation energy at this time being 2.828 eV. This calculated CT effect robustly explains the concentration-dependent fluorescence quenching observed in our experiments (Figures 6D,E). As Phide molecules parallelly stack into aggregates, these CT states open efficient non-radiative decay pathways that dissipate destructive excitation energy, thereby fundamentally preventing photo-degradation and enhancing overall color stability. The lower excitation energy required by Phide indicated a greater likelihood of CT effects occurring. It can thus be surmised that electroinduced molecular aggregation may have a greater effect on Phide. The method of improving the stability of Phide through electroinduced aggregation is worthy of further study.

## 4 Conclusion

This study successfully synthesized Phide, a natural water-soluble derivative of Chl, with safety and ease. Spectroscopic, FT-IR, and colorimetric analyses demonstrated that the Phide structure lacks a phytyl chain. This created novel applications for natural green pigments in food processing. This study exhibited a straightforward, and precise method for quantifying Phide concentration. The HPLC-UV–Vis spectral method for determining Phide concentration is effective. The absorption measurement of the sample’s UV–Vis spectrum at 418 nm provided a formula for Phide concentration. This study established that aggregation induced by high concentrations enhances the stability of Phide in aqueous solution. Quantum chemistry calculations indicated that Phide readily forms dimers in aqueous solution, primarily through hydrogen bonding as the dominant interaction force. The charge transfer effects of Chl and Phide in aqueous solution indicated that Phide exhibited reduced excitation energy, enhanced charge transfer propensity, and increased aggregate stability. In conclusion, this research not only offers a simple formula for Phide quantification, *C*_Phide_ (mg/L) = 4.726 * A_418_, but also demonstrates that molecular aggregation is a viable strategy for protecting sensitive natural pigments. Transitioning from this model system to complex food applications will be the next critical step in validating the commercial potential of Phide as a safer alternative to synthetic dyes.

In this study, In order to meet the practical needs, we did not conduct a detailed structural analysis of the saponification products of chlorophyll. To overcome the shortcomings of the present research, future work could employ techniques such as nuclear magnetic resonance (NMR) and mass spectrometry (MS) for detailed structural analysis of the products.

## Data Availability

The original contributions presented in the study are included in the article/Supplementary material, further inquiries can be directed to the corresponding authors.
