# Supplementary material for: Aggregation-induced stabilization of pheophorbide, a water-soluble chlorophyll derivative
Source: Front Nutr. 2026 May 15;13:1847974. doi: 10.3389/fnut.2026.1847974 (PMC13218883; doi:10.3389/fnut.2026.1847974)
Supplement: Supplementary file 1 [file Table_1.DOCX]

Supporting Information

Aggregation-Induced Stabilization of Pheophorbide, a Water-Soluble Chlorophyll Derivative

Yixiao Liu^a 1^, Yishuang Liu^a 1^, Yangbin Wang^a^, Shuyu Wang^a^, Shuyi Shi^b,c^, Fangwei Li^a,*^, Mingyong Zeng^a,^[[1]](#footnote-0)^*^

^a^ State Key Laboratory of Marine Food Processing and Safety Control, College of Food Science and Engineering, Ocean University of China, Qingdao 266404, China / Sanya Institute of Oceanography, Ocean University of China, Sanya 572000, China.

^b^ College of Food Science and Nutritional Engineering, China Agricultural University, Beijing 100083, People's Republic of China.

^c^ National Engineering Research Center for Fruit and Vegetable Processing, Ministry of Science and Technology, Beijing 100083, People's Republic of China.

**Table of Contents**

[1. Tables 3](#_Toc79948365)

1. Tables

**Table S1.** Orbital contribution of Chl dimer S_1_-S_5_.

| Excited  states | Excitation  energy  (eV) | Orbitals | Number of  occupied  electrons | Contribution  rate to holes  （%） | Contribution  rate to  electrons（%） |
| --- | --- | --- | --- | --- | --- |
| S_1_ | 2.060 | 479 | 2 | 6.516 | 0.000 |
|  |  | 480 | 2 | 4.489 | 0.000 |
|  |  | 481 | 2 | 51.021 | 0.000 |
|  |  | 482（HOMO） | 2 | 36.226 | 0.000 |
|  |  | 483（LUMO） | 0 | 0.000 | 51.210 |
|  |  | 484 | 0 | 0.000 | 36.272 |
|  |  | 485 | 0 | 0.000 | 6.037 |
|  |  | 486 | 0 | 0.000 | 4.240 |
| S_2_ | 2.069 | 479 | 2 | 4.721 | 0.000 |
|  |  | 480 | 2 | 6.777 | 0.000 |
|  |  | 481 | 2 | 35.983 | 0.000 |
|  |  | 482（HOMO） | 2 | 50.771 | 0.000 |
|  |  | 483（LUMO） | 0 | 0.000 | 36.049 |
|  |  | 484 | 0 | 0.000 | 50.995 |
|  |  | 485 | 0 | 0.000 | 4.418 |
|  |  | 486 | 0 | 0.000 | 6.231 |
| S_3_ | 2.427 | 479 | 2 | 74.376 | 0.000 |
|  |  | 481 | 2 | 23.357 | 0.000 |
|  |  | 483 | 0 | 0.000 | 76.025 |
|  |  | 485 | 0 | 0.000 | 21.962 |
| S_4_ | 2.431 | 480 | 2 | 73.949 | 0.000 |
|  |  | 482 | 2 | 23.774 | 0.000 |
|  |  | 484 | 0 | 0.000 | 75.598 |
|  |  | 486 | 0 | 0.000 | 22.399 |
| S_5_ | 3.357 | 482 | 2 | 99.967 | 0.000 |
|  |  | 483 | 0 | 0.000 | 99.678 |

**Table S2.** Orbital contribution of Phe dimer S_1_-S_5_.

| Excited  states | Excitation  energy(eV) | Orbitals | Number of  occupied  electrons | Contribution  rate to holes（%） | Contribution  rate to  electrons（%） |
| --- | --- | --- | --- | --- | --- |
| S_1_ | 2.057 | 303 | 2 | 6.802 | 0.000 |
|  |  | 304 | 2 | 7.273 | 0.000 |
|  |  | 305 | 2 | 39.383 | 0.000 |
|  |  | 306（HOMO） | 2 | 44.792 | 0.000 |
|  |  | 307（LUMO） | 0 | 0.000 | 46.022 |
|  |  | 308 | 0 | 0.000 | 38.085 |
|  |  | 309 | 0 | 0.000 | 7.004 |
|  |  | 310 | 0 | 0.000 | 6.591 |
| S_2_ | 2.100 | 303 | 2 | 9.308 | 0.000 |
|  |  | 304 | 2 | 8.987 | 0.000 |
|  |  | 305 | 2 | 40.200 | 0.000 |
|  |  | 306（HOMO） | 2 | 39.680 | 0.000 |
|  |  | 307（LUMO） | 0 | 0.000 | 40.331 |
|  |  | 308 | 0 | 0.000 | 39.513 |
|  |  | 309 | 0 | 0.000 | 8.828 |
|  |  | 310 | 0 | 0.000 | 8.729 |
| S_3_ | 2.439 | 303 | 2 | 36.700 | 0.000 |
|  |  | 304 | 2 | 35.278 | 0.000 |
|  |  | 305 | 2 | 14.965 | 0.000 |
|  |  | 306（HOMO） | 2 | 11.456 | 0.000 |
|  |  | 307（LUMO） | 0 | 0.000 | 45.079 |
|  |  | 308 | 0 | 0.000 | 27.921 |
|  |  | 309 | 0 | 0.000 | 14.690 |
|  |  | 310 | 0 | 0.000 | 10.653 |
| S_4_ | 2.451 | 303 | 2 | 32.608 | 0.000 |
|  |  | 304 | 2 | 35.733 | 0.000 |
|  |  | 305 | 2 | 12.685 | 0.000 |
|  |  | 306（HOMO） | 2 | 17.356 | 0.000 |
|  |  | 307（LUMO） | 0 | 0.000 | 29.777 |
|  |  | 308 | 0 | 0.000 | 40.096 |
|  |  | 309 | 0 | 0.000 | 12.788 |
|  |  | 310 | 0 | 0.000 | 15.656 |
| S_5_ | 2.828 | 305 | 2 | 31.130 | 0.000 |
|  |  | 306（HOMO） | 2 | 67.578 | 0.000 |
|  |  | 307（LUMO） | 0 | 0.000 | 64.347 |
|  |  | 308 | 0 | 0.000 | 35.223 |

1. ^1^ These authors contributed equally to this work.

   * Corresponding author. E-mail address: lifangwei@ouc.edu.cn (Fangwei Li); [mingyz@ouc.edu.cn](mailto:mingyz@ouc.edu.cn) (Mingyong Zeng). [↑](#footnote-ref-0)
